# Intraocular endoscopy for the evaluation and treatment of hypotony due to a traumatic cyclodialysis: a case report

**DOI:** 10.1186/s12886-020-01375-3

**Published:** 2020-03-23

**Authors:** Henry Bair, Chun-Ju Lin, Chun-Ting Lai, Ning-Yi Hsia, Yi-Yu Tsai

**Affiliations:** 1 Department of Ophthalmology, China Medical University Hospital, China Medical University, 2 Yuh-Der Road, Taichung City, 40447 Taiwan; 2 Stanford University School of Medicine, Stanford, CA USA; 3 School of Medicine, College of Medicine, China Medical University, Taichung, Taiwan; 4 Department of Optometry, Asia University, Taichung, Taiwan

**Keywords:** Cyclodialysis, Intraocular endoscopy, Traumatic hypotony

## Abstract

**Background:**

A cyclodialysis cleft often leads to direct communication between the anterior chamber and the suprachoroidal space. It is a rare condition that is encountered with blunt trauma, and less commonly, after surgery. Hypotony is the major sequelae that may lead to hypotonous maculopathy, optic disc edema, corneal folds, and astigmatism. These may cumulatively lead to visual loss. We describe how endoscopy in a cyclodialysis repair allowed us to accurately locate the cleft and guided its appropriate management avoiding unnecessary cryopexy.

**Case presentation:**

A 41-year-old male experienced a traumatic cyclodialysis cleft, which resulted in persistent hypotony. Pars plana vitrectomy was performed to treat vitreous hemorrhage. Scleral indentation was attempted to visualize the cyclodialysis cleft. However, the depression distorted the visualization. Intraocular endoscopy was therefore used to evaluate the cleft. Guided by this assessment, only intraocular gas tamponade was used to reposition the ciliary body. The patient’s intraocular pressure was restored to 13 mmHg 3 days after the operation, and OCT confirmed cleft closure 1 month after the operation.

**Conclusion:**

Endoscopy-assisted repair of cyclodialysis is an approach that enhances visualization and can guard against common causes of persistent cleft and hypotony, as well as reveal the causes of recurrent failure. Hence, it can eliminate unnecessary cryopexy that might worsen the hypotonous state. In our case, intraocular endoscopy was effective for the evaluation of a cyclodialysis cleft and the subsequent selection of an appropriate management technique, gas tamponade, that was more conservative than other approaches initially considered.

## Background

A cyclodialysis cleft often leads to direct communication between the anterior chamber (AC) and the suprachoroidal space [1, 2]. It is a rare condition that is encountered with blunt trauma [3], and less commonly, after surgery [4]. Hypotony is the major sequelae that may lead to hypotonus maculopathy, optic disc edema, corneal folds, and astigmatism. These may cumulatively lead to visual loss [5]. In examining a suspected cyclodialysis cleft, variable findings and difficult visualization by gonioscopy through a soft globe and edematous cornea often necessitate the use of ultrasound biomicroscopy (UBM) or anterior segment optical coherence tomography (OCT) [1, 6].

Smaller clefts may spontaneously close, especially with the assistance of medications or apposition of the ciliary body (CB) and scleral spur (SS) using gas tamponade or a temporary scleral buckle [2, 7]. Nonetheless, visualization is a major concern, especially in the context of traumatic effects, including corneal edema and hyphema [8]. Visualization during procedures can present especially challenging scenarios (since neither UBM nor anterior segment OCT are used as intraoperative examination tools). In this report, we describe how endoscopy used intraoperatively in a cyclodialysis repair allowed us to accurately locate the cleft and guided its appropriate management avoiding unnecessary cryopexy.

## Case presentation

**Fig. 1:**
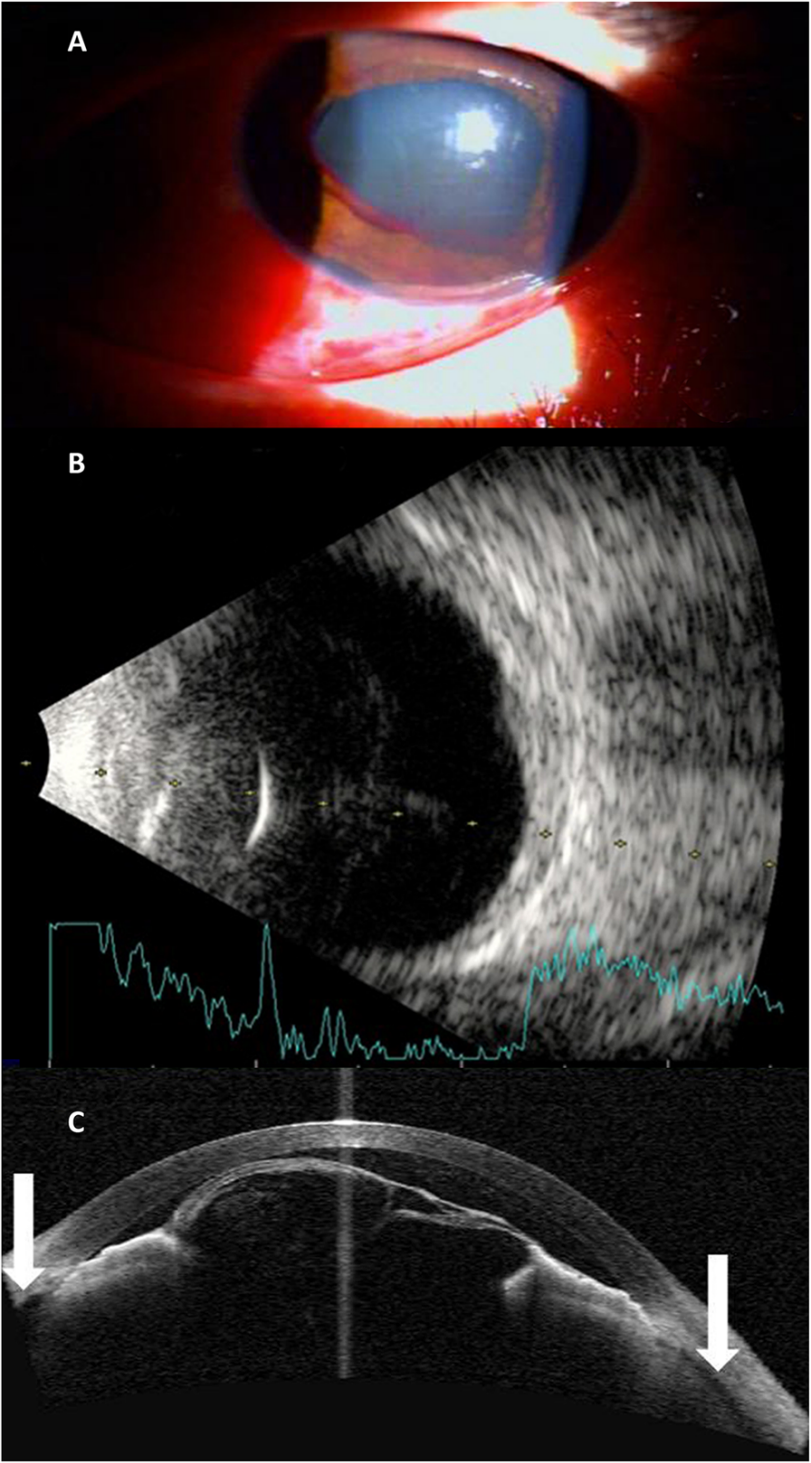
**a** Marked conjunctival hemorrhage, hyphema, mydriatic pupil, lens anterior dislocation, iridodialysis, vitreous prolapse, and hemorrhage were noted OS. **b** B scan showed dense vitreous hemorrhage without obvious retinal detachment. **c** Anterior OCT showed shallow AC, anteriorly dislocated lens, and supraciliary effusion (white arrow)

A 41-year-old male without significant medical, family, and psychosocial history presented to our service 1 day after suffering a work-related projectile injury with severely decreased vision in the left eye. His best corrected visual acuity (BCVA) was 20/20 OD and counting fingers OS. Intraocular pressure (IOP) was 3 mmHg OS. Marked conjunctival hemorrhage, hyphema, mydriatic pupil, anterior lens dislocation, iridodialysis, and vitreous prolapse and hemorrhage were noted OS (Fig. 1a). B-scan showed dense vitreous hemorrhage without retinal detachment (Fig. 1b). Anterior OCT (Visante OCT™, Carl Zeiss, Germany) revealed a shallow anterior chamber, anteriorly dislocated lens, and a supraciliary effusion OS (Fig. 1c). The right eye was unremarkable on examination.

**Fig. 2:**
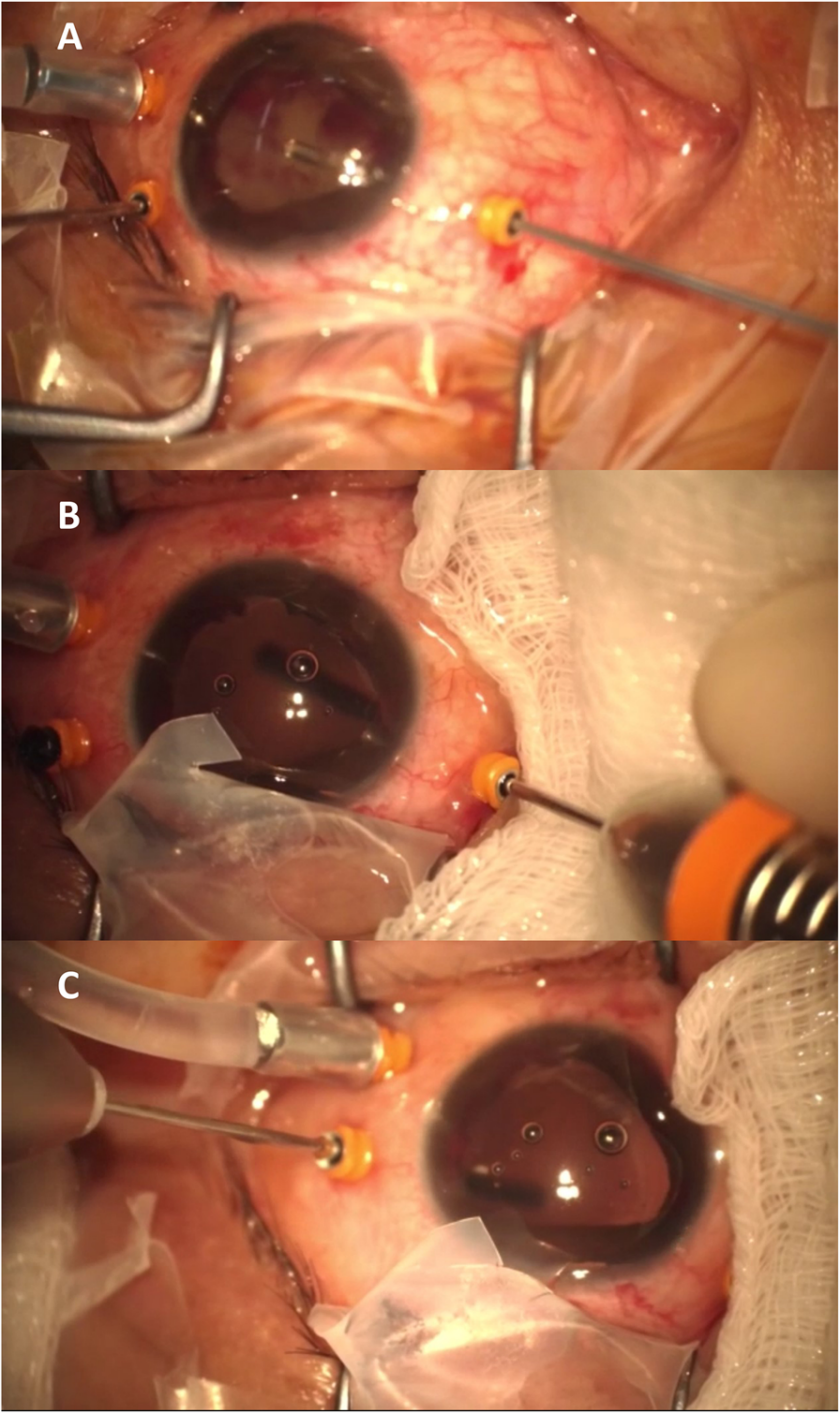
**a** Lensectomy and 23-gauge vitrectomy with 6 mm infusion cannula. **b** and **c** Intraocular endoscope performed through the 23-gauge valved trocar used for the initial lensectomy and vitrectomy

**Fig. 3:**
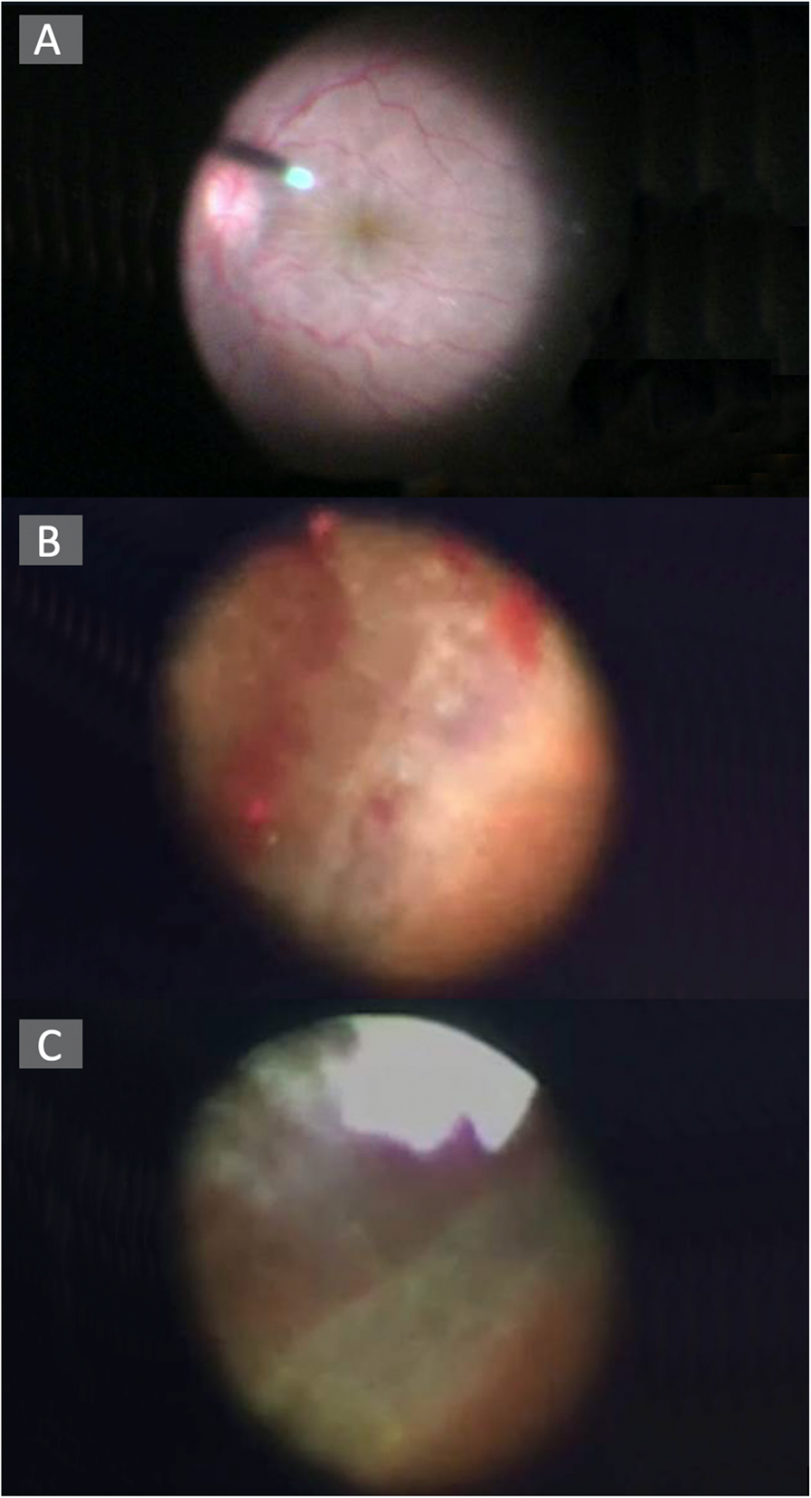
**a** Hypotony maculopathy was found after removing the dense vitreous hemorrhage. **b** Intraocular endoscopy demonstrated only localized traction. **c** Some whitish material on the ciliary processes were found by intraocular endoscopy

A 10-day course of topical and systemic steroids could not resolve the hypotony. Thus, lensectomy and 23-gauge vitrectomy with 6 mm infusion cannula (Fig. 2a) were performed using the Resight wide-angle viewing system. Hypotony maculopathy was detected after removing the dense vitreous hemorrhage (Fig. 3a). A cyclitic membrane was identified, then peeled completely. Scleral indentation was attempted to examine the status of the ciliary body, but the depression distorted the visualization. An intraocular endoscope was therefore used via the original 23 g valved trocar (Fig. 2b, c) for evaluation, and found a cyclodialysis cleft extending from the 10 to 2 o’clock positions. Only localized traction (Fig. 3b) and some whitish fibrous material on the ciliary processes (Fig. 3c) were seen. The fibrous tissue was dissected and only intraocular gas endotamponade (20% SF6) was performed to reposition the ciliary body without using cryopexy. Left IOP was restored to 13 mmHg 3 days after the surgery.

**Fig. 4:**
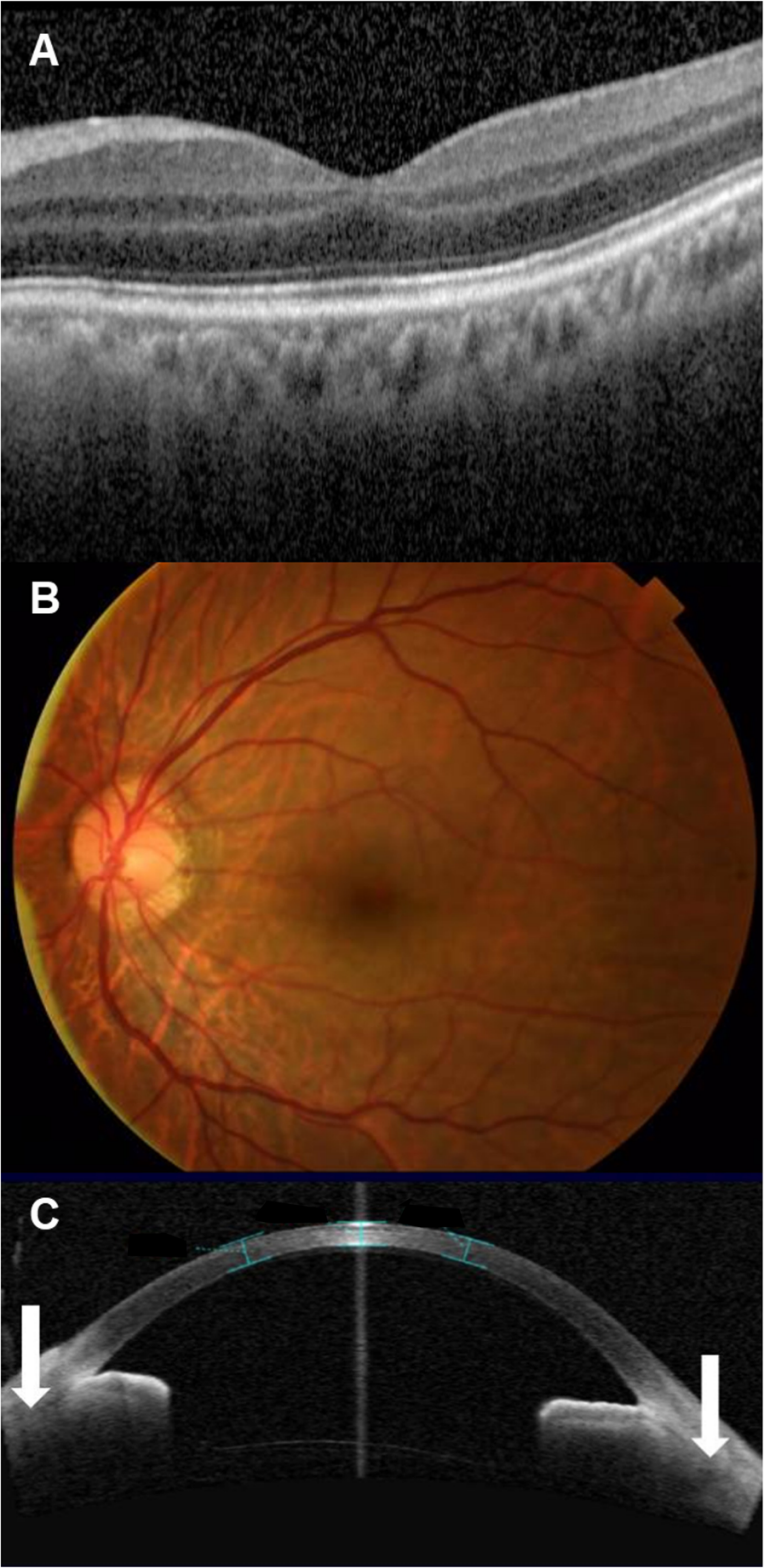
**a** The OCT showed no obvious hypotony maculopathy. **b** There was no more hypotony maculopathy 2 months postoperatively. **c** Anterior OCT showed cleft closure (white arrow) and suture PCIOL in the proper position

One month after the surgery, the patient’s IOP remained at 12 mmHg and OCT showed no obvious hypotony maculopathy (Fig. 4a), which was completely resolved after 2 months (Fig. 4b). After that, a sutured posterior chamber intraocular lens (PCIOL) was arranged. Anterior OCT confirmed cleft closure and proper positioning of the sutured PCIOL (Fig. 4c). His IOP remained 12 mmHg after 18 months, and BCVA improved to 20/200.

## Discussion and conclusions

A cyclodialysis cleft occurs due to the separation of the ciliary muscles from the scleral spur, opening a direct path for the aqueous humor to the suprachoroidal space [1, 2]. Vision can be severely impaired due to AC shallowing, chorio-retinal folds, and hypotony maculopathy. Gonioscopy is essential on clinical suspicion; however, in traumatic events, it is difficult to rely on, and imaging techniques such as UBM or anterior segment OCT may be warranted [1, 6].

Spontaneous closure appears more likely with small clefts. For larger clefts, different therapeutic plans have been proposed and discussed, ranging from conservative treatments such as topical atropine to dilate the pupil and hence appose the CB and SS, allowing natural inflammatory processes to close the wound, to laser photocoagulation [5]. Direct cyclopexy is a simple and effective technique for repair. Full-thickness suturing under the scleral flap has demonstrated much success, with restoration of normal IOP after the repair [5, 9]. Different modifications have been introduced with comparable outcomes.

Visualization of the cleft has always been a concern. In many cases, while the cleft appeared to be closed intraoperatively, postoperative persistent hypotony led to reimaging that revealed persistent clefts [2]. This has been attributed to insufficient laser ablation or incomplete penetrance of the sutures. In these instances, endoscopic photocoagulation could have enhanced reattachment rates. However, intraoperative endoscopy has not previously been thoroughly evaluated in cyclodialysis repair.

In our case, the cleft was the result of projectile trauma, and was accompanied by hyphema, anterior lens dislocation, and vitreous hemorrhage. After medical therapy failed to close the cleft, pars plan lensectomy and vitrectomy with peeling of cyclitic membrane was performed, and the CB was assessed. Scleral indentation distorted the view of the CB, and endoscopy revealed localized CB traction with whitish material on ciliary processes. The fibrous membranes were peeled off, and gas tamponade was performed. Endoscopy then revealed the cleft to be small enough that no suturing or laser ablation was warranted, thus avoiding unnecessary cryopexy which might cause further CB damage and subsequent impairment of aqueous humor production. IOP was restored to its normal levels and vision was improved to 20/200. Residual visual impairment may be attributed to the sequelae of the previous hypotony maculopathy.

We conducted a literature search to find similar reports that used such an approach in traumatic cyclodialysis with hypotony; however, we could not find any relevant articles. A similar concept was reported by Gnanaraj et al. [10], who employed endoscopy-assisted repair with similar success. However, Gnanaraj et al. employed endoscopy in a 4-year-old girl with post-goniotomy cyclodialysis after failure of all other approaches, including suturing and cryoablation of CB. Endoscopy revealed incomplete penetrance of the sutures and allowed suturing under direct visualization with longer needles. UBM confirmed an attached CB, and BCVA stabilized at 20/200 due to amblyopia [10].

Endoscopy-assisted repair of cyclodialysis is an approach that enhances visualization and can guard against common, yet hidden, causes of persistent cleft and hypotony (such as localized traction with fibrous membrane in our case), or reveal the causes of recurrent failure (partial suture pass as in Gnanaraj et al. [10]). Hence, it can eliminate potentially unnecessary cryopexy, which might worsen the hypotonous state. In our case, endoscopy allowed us to determine that gas tamponade would be the most efficacious course of action to close the cleft, rather than cryopexy or suturing, which may have promoted intense inflammatory response or rebleeding, was not essential. Furthermore, endoscopy, an invasive procedure, was particularly facilitated in our case by the fact that we could insert the probe through the 23 g valved trocar used for the initial lensectomy and vitrectomy, thus avoiding the necessity of a second wound. This raises an important limitation of the use of endoscopy in cyclodialysis cleft repairs, in that endoscopy is unsuitable for phakic patients, as there would be a possibility of precipitating cataracts. Additional considerations include the inherent difficulty in orienting the field of view and the substantial dexterity required to maneuver the endoscope. It is also important to note that endoscopy cannot be used during surgeries requiring bimanual techniques.

In conclusion, we propose that endoscopy can be considered in cases of cyclodialysis repair involving complications such as anterior lens dislocation, iridodialysis, and vitreous hemorrhage, and involving procedures such as pars plana lensectomy and vitrectomy. More cases are needed to allow better evaluation and modifications of this technique.

## Data Availability

All data generated or analyzed during this study are included in this published article.

## Abbreviations

AC: Anterior chamber
UBM: Ultrasound biomicroscopy
OCT: Optical coherence tomography
CB: Ciliary body
SS: Scleral spur
IOP: Intraocular pressure
BCVA: Best corrected visual acuity
PCIOL: Posterior chamber intraocular lens
